# Ethylenediurea (EDU) effects on Japanese larch: an one growing season experiment with simulated regenerating communities and a four growing season application to individual saplings

**DOI:** 10.1007/s11676-020-01223-6

**Published:** 2020-09-30

**Authors:** Evgenios Agathokleous, Mitsutoshi Kitao, Xiaona Wang, Qiaozhi Mao, Hisanori Harayama, William J. Manning, Takayoshi Koike

**Affiliations:** 1 Key Laboratory of Agrometeorology of Jiangsu Province, Institute of Applied Ecology, Nanjing University of Information Science and Technology (NUIST), Nanjing, 210044 People’s Republic of China; 2 Division of Environment and Resources Research, Research Faculty of Agriculture, Hokkaido University, Sapporo, 060-8589 Japan; 3 Hokkaido Research Center, Forestry and Forest Products Research Institute (FFPRI), Sapporo, 062-8516 Japan; 4 College of Landscape Architecture and Tourism, Hebei Agricultural University, No. 2596 Lekai South Street, Lianchi District, Baoding, 071000 People’s Republic of China; 5 College of Resources and Environment, Southwest University, Chongqing, 400700 People’s Republic of China; 6 Ecophysiology Laboratory, Department of Plant Ecology, Forestry and Forest Products Research Institute (FFPRI), Matsunosato-1, Tsukuba, 305-8687 Japan; 7 Department of Plant, Soil and Insect Sciences, University of Massachusetts, 80 Campus Center Way, Amherst, MA 01003 USA; 8 Shenzhen Graduate School of Environment and Energy, Peking University, Shenzhen, 518055 People’s Republic of China; 9 Research Center for Eco-Environmental Science, Chinese Academy of Science, Beijing, 100085 People’s Republic of China

**Keywords:** Air pollution, Antiozonant, Ethylenediurea (EDU), Plant protection, Tropospheric ozone (O_3_)

## Abstract

Japanese larch (*Larix kaempferi* (Lamb.) Carr.) and its hybrid are economically important coniferous trees widely grown in the Northern Hemisphere. Ground-level ozone (O_3_) concentrations have increased since the pre-industrial era, and research projects showed that Japanese larch is susceptible to elevated O_3_ exposures. Therefore, methodologies are needed to (1) protect Japanese larch against O_3_ damage and (2) conduct biomonitoring of O_3_ in Japanese larch forests and, thus, monitor O_3_ risks to Japanese larch. For the first time, this study evaluates whether the synthetic chemical ethylenediurea (EDU) can protect Japanese larch against O_3_ damage, in two independent experiments. In the first experiment, seedling communities, simulating natural regeneration, were treated with EDU (0, 100, 200, and 400 mg L^−1^) and exposed to either ambient or elevated O_3_ in a growing season. In the second experiment, individually-grown saplings were treated with EDU (0, 200 and 400 mg L^−1^) and exposed to ambient O_3_ in two growing seasons and to elevated O_3_ in the succeeding two growing seasons. The two experiments revealed that EDU concentrations of 200–400 mg L^−1^ could protect Japanese larch seedling communities and individual saplings against O_3_-induced inhibition of growth and productivity. However, EDU concentrations ≤ 200 mg L^−1^ did offer only partial protection when seedling communities were coping with higher level of O_3_-induced stress, and only 400 mg EDU L^−1^ fully protected communities under higher stress. Therefore, we conclude that among the concentrations tested the concentration offering maximum protection to Japanese larch plants under high competition and O_3_-induced stress is that of 400 mg EDU L^−1^. The results of this study can provide a valuable resource of information for applied forestry in an O_3_-polluted world.

**Electronic supplementary material:**

The online version of this article (10.1007/s11676-020-01223-6) contains supplementary material, which is available to authorized users.

## Introduction

Larches (Pinaceae) are deciduous conifers of remarkable ecological and economic value, with a widespread existence and growth throughout the Northern Hemisphere, including a dominant role in the community structure of the boreal forests of Siberia and Canada (Farjon 1990; Abaimov et al. 2000; Osawa et al. 2010). Japanese larch (*Larix kaempferi* (Lamb.) Carr.) is an important timber species for wood production in Asia as well as in Europe, where its wood has been used for multiple purposes such as railway sleepers, construction, pit props and pulp industry (Ryu et al. 2009; Kurinobu 2015; Da Ronch et al. 2016). For instance, Japanese larch is a major plantation species in Japan, and has a breeding history of about 70 years (Kurinobu 2015). However, its breeding has focused on the improvement of wood quality and resistance to pests and diseases (e.g. needle cast disease) so far (Ryu et al. 2009; Kurinobu 2015), and Japanese larch and its hybrids have been found susceptible to ground-level ozone (O_3_), with often suppressed plant size and reduced biomass at elevated concentrations (Koike et al. 2012; Wang et al. 2015; Agathokleous et al. 2017; Sugai et al. 2018).

O_3_ is a greenhouse gas forming through the reaction of its precursors (mainly volatile organic compounds and NOx) under high ultraviolet light (Saitanis et al. 2020). Average daytime O_3_ levels, when plants are fully functioning physiologically and ‘breathe’ most O_3_, have increased since the industrial revolution, and remain elevated in rural and urban areas throughout the Northern Hemisphere nowadays (Chang et al. 2017; Schultz et al. 2017; Mills et al. 2018). In general, based on vegetation exposure indices, the highest O_3_ concentrations occur in mid-latitudes including north, north-west and east China, the Republic of Korea, and Japan (Mills et al. 2018). These regions happen to be major habitats of the Japanese larch too, suggesting a potential risk for O_3_-induced damage (Hoshika et al. 2020). The complex chemistry of O_3_ and its trans-boundary transport make difficult its control (Ganev et al. 2008; Kleanthous et al. 2014; Gao et al. 2020), and predictions for 2100 indicate that O_3_ may remain at potentially phytotoxic levels even with the most optimistic 2100 climate scenario (Sicard et al. 2017). The difficulty in decreasing O_3_ levels is further documented by a recent study showing that, while many other air pollutants decreased, O_3_ levels have considerably increased in cities due to the COVID-19 lockdown imposed in 2020 (Sicard et al. 2020). For these reasons, research is needed to invent methodologies to protect Japanese larch and other tree species against O_3_ damage.

Several research projects have been directed to protect plants against O_3_ damage, by using various methods or approaches: (1) introducing symbiotic microbes into the plant system to mediate plant response (Agathokleous et al. 2020), (2) modifying the irrigation management practices to reduce O_3_ uptake from stomata (Harmens et al. 2019), (3) applying substances on leaf surface to create a protective “membrane” and prevent O_3_ from entering the leaf tissue (Agathokleous et al. 2016b), and (4) treating plants with chemicals that systemically protect plants against O_3_ damage (Tiwari 2017). However, only approach (4) has been widely researched and shown to produce sufficient protection. In spite of the many chemical compounds tested in the framework of approach 4, the largest research effort has been placed on ethylenediurea (EDU) (Tiwari 2017). EDU sufficiently protected a wide array of plant species and numerous cultivars and genotypes against O_3_ damage (Feng et al. 2010; Manning et al. 2011; Oksanen et al. 2013; Singh et al. 2015; Agathokleous 2017; Chaudhary and Rathore 2020). The vast majority of these studies concerns cultivated crop plants, while relatively few studies concern broadleaved tree species. A literature survey suggests that whether EDU can protect coniferous trees against O_3_ damage has not been tested to date. The EDU mode of action in protecting plants against O_3_ damage remains unclear (Singh et al. 2015; Agathokleous 2017; Tiwari 2017; Gupta et al. 2020). However, it appears that EDU protects plants within a hormetic framework, by acting as a xenobiotic to activate defense mechanisms at low doses, in contrast to adverse effects at high doses (Wang et al. 2007; Agathokleous 2017; Salvatori et al. 2017; Tiwari 2017; Gupta et al. 2018; Xu et al. 2019); however, reactions on the leaf surface also occur and partly reduce some O_3_ effect (Ashrafuzzaman et al. 2018).

For the first time, this study aims at assessing whether EDU can protect Japanese larch against O_3_ damage, in two independent experiments. In the first experiment, a dose–response evaluation was conducted to study EDU effects (4 concentrations) on Japanese larch communities of generating seedlings under ambient or elevated O_3_, with the aim to identify the EDU concentration offering the highest protection against O_3_ damage. A unique feature of this experiment is that it simulates regenerating highly dense communities of Japanese larch, as previous experiments studying EDU effects on tree species included individual trees with no intraspecific competition (Paoletti et al. 2009; Agathokleous 2017; Xu et al. 2019). The only EDU experiment with mesocosms was with manna ash (*Fraxinus ornus* L.); however, saplings were planted with a low density and subjected to only ambient O_3_, and the experiment was directed to assess the temporal variation in physiology but not effects on growth and productivity (Salvatori et al. 2017). Therefore, this is the first study testing whether growth and productivity of communities of a tree species can be benefited from EDU in an O_3_-polluted atmosphere. In the second experiment, the research question was whether EDU can offer long-term protection of Japanese larch plants against O_3_ damage. To this end, individually-grown saplings were exposed to ambient O_3_ for 2 years and to elevated O_3_ for 2 more years (4 years in total), while treated with EDU (3 concentrations). Nearly all the published studies of EDU effects on plants lasted for only one growing season, while only one experiment with a fast-growing O_3_-susceptible hybrid poplar lasted for several years (Hoshika et al. 2013; Katanić et al. 2014; Carriero et al. 2015; Giovannelli et al. 2019). Therefore, this experiment with Japanese larch is of high value for potential forestry applications in the future.

## Materials and methods

### Study site and plant materials

Two independent experiments were conducted in the experimental nursery of Sapporo Experimental Forest, Field Science Center (FSC) of Hokkaido University, Sapporo, Japan (43°0′ N, 141°2′ E, 15 m a.s.l.), where the snow-free period lasts from April to November. The model plant was Japanese larch: *L. kaempferi* (Lamb.) Carr. for both experiments. Meteorological data were recorded by a nearby station at Sapporo (43°03.6′ N 141°19.7′ E), which is monitored by the Japan Meteorological Agency (http://www.jma.go.jp/jma/indexe.html).

### Experiment I

This experiment was conducted in 2017 and lasted for one growing season. In contrast to previous experiments with EDU applied to various tree species, seedlings were grown under high intraspecific competition, so to experience elevated O_3_ under the pressure of high competition at very early ontogenic stages (soon after regeneration) with expected relatively higher susceptibility.

#### Plant material preparation

In this experiment, seeds were germinated in Petri dishes under laboratory conditions. More information about the origin and seed treatment can be found in (Agathokleous et al. 2020).

On 7 June, homogenous Petri-grown seedlings were selected for experimentation, and the root length and total germinated seed fresh weight of 30 randomly selected seedlings were measured with a measuring tape (1 mm accuracy) and an electronic scale (3 decimal accuracy). The average root length was 1.44 ± 0.08 (± hereafter indicates standard error, s.e., unless specified otherwise) cm and the average germinated seed fresh weight was 15.73 ± 1.31 mg. Twenty-four plastic pots (5-L each) were filled with coconut peat (topcocopeat, Top, Osaka, Japan), and irrigated well. The pots were placed (completely randomized design) in a glasshouse of Hokkaido University (43°04′ N, 141°20′ E, 15 m a.s.l.); the windows were kept open, and the environment uncontrolled. The position of the pots was randomly rotated every other day. On 8 June, 15 seedlings were transplanted in each pot (360 seedlings in total) to simulate regeneration with intraspecific competition (density = 433 seedlings m^−2^). A dose of 6 g of slow-release fertilizer [Osmocote Exact Standard 8–9 M (15-9-11 + 2MgO + TE), Hyponex Japan Corp. Ltd, Osaka, Japan] was added to each pot. This is a low dose for 15 seedlings, considering that a dose of 2 g given to single plants grown in 0.2-L pots had significant fertilizing effect on Japanese larch plants compared to single plants grown in 0.2-L pots containing 1 g of this fertilizer (Agathokleous et al. 2020). However, the dose used was sufficient for normal growth of the seedlings in the presence of high competition, and there was no observable sign of stress induced by nutrient deficiency over the experimental period.

#### EDU treatments

On 9 June, a dose of 200 mL of water solution containing 0 (EDU0), 100 (EDU100), 200 (EDU200) or 400 (EDU400) mg L^−1^ EDU was added to each pot (treatments assigned randomly). Pure water was used for all the treatments. The equally spaced concentrations were selected based on the existing literature showing that EDU positive effects as well as potentially the hormetic zone (i.e. the zone with stimulatory effects in a dose–response relationship; below the toxicological threshold) occur within this concentration range (Agathokleous 2017). Each pot was placed in a plate before treatments, and the run-off water was given back to the pots for irrigation with additional water if needed throughout the experiment; the soil water was maintained at field capacity throughout the experiment. The treatments were repeated every 9 days, according to a literature analysis (Agathokleous 2017), for a total of 11 applications. For all the treatments and application times, treatment solutions were prepared freshly 30–60 min before application. All EDU soil drench treatments conducted during late afternoon to evening (16:00–18:00). The pots were rotated within and across experimental units of the same O_3_ treatment every approximately 10 days.

#### O_3_ treatments

On 17 June, pots were moved to the experimental nursery, where a free-air O_3_-concentration enrichment (FACE) system with an early-successional community of trees operated. This system has been used for a number of published studies (Agathokleous et al. 2017), and a detailed description is provided in Supplementary Information made available online along with this paper. During June–September (experimental months), the climatic conditions were (monthly mean ± S.D.): daily average air temperature = 19.58 ± 3.26 °C, daily maximum air temperature = 24.18 ± 3.16 °C, daily minimum air temperature = 15.90 ± 3.54 °C, wind speed = 3.10 ± 0.24 m s^−1^, relative humidity = 70.50 ± 2.65%, total sunshine duration = 183.58 ± 14.30 h, and total precipitation = 127.38 ± 58.67 mm.

Pots were allocated to the six O_3_ plots during late evening. Three of the plots were ambient O_3_ (AOZ) and the other three were ambient O_3_ enriched with additional O_3_ to simulate an elevated O_3_ exposure (EOZ). In each O_3_ plot, 4 pots, 1 per EDU treatment, were randomly selected and placed at the north side of the plot (the design of the O_3_ plots is illustrated in Figs. S1–S3, Supplementary Information). EOZ treatment was performed during the daytime, on a daily basis, and lasted from June 18 (day of second EDU application) to September 18.

Ambient O_3_ concentrations were logged by an ultraviolet absorption O_3_ analyzer (TUV-1100; Tokyo Industries Inc. Tokyo, Japan) every 1 min (for details see Supplementary Information). The average daily 10-h (08:00–18:00 Japan Standard Time, JST) concentration of ambient O_3_, during the O_3_ exposure period, was 39.8 ± 7.6 (s.d.) nmol mol^−1^. For the same period, the average daily 10-h (07:00–17:00 JST) concentration of O_3_ in the EOZ treatment was 57.5 ± 8.5 (s.d.) nmol mol^−1^, i.e. ~ 1.5 times higher than the ambient O_3_ concentration. The AOT40 values were 5.0 μmol mol^−1^ for AOZ and 18.3 μmol mol^−1^ for EOZ.

#### Data collection

At the end of the experiment, growth and biomass were measured in all plants. Stem diameter was measured with a digital caliper (0.01-mm grading) at the stem base (2 cm height) as the average of two crosswise measurements. Shoot height was measured with a measuring tape (1-mm grading) as the distance from the bottom to the top of the shoot. Crown span was measured as the distance between the two farthest points of the crown, as the average of two crosswise measurements. Crown depth was measured as the distance from the bottom to the top of crown. Shoot height, crown span, and crown depth were measured with a measuring tape (1-mm grading). Seedlings were removed intact from the soil substrate, and the roots were gently washed with tap water to remove remaining soil. Root length was measured with a measuring tape (1-mm grading). Seedlings were separated into needles, stems, and roots, and air-dried in an oven (65 °C) until constant mass. Dry mass of each segment of all plants was measured with an electronic scale (3-decimal accuracy), and the total biomass per plant community (per pot) per plant segment was calculated as the sum of all seedlings.

### Experiment II

This experiment was conducted over 4 growing seasons (2014–2017). EDU was applied to individually-grown saplings.

#### Plant material preparation

In this experiment, saplings were used. Two-year old saplings of Japanese larch, which were kept in low temperature under dark, were offered from the nursery of Forestry Research Institute of Hokkaido Research Organization at Bibai city near Sapporo (saplings were obtained from trees growing under full sunlight). Saplings were moved to Hokkaido University, Sapporo campus, on May 15, 2014, and stored in an incubator at 3 °C (± 0.5 °C) under light. On 5 June, each of 23 uniform saplings was planted into a 15-L plastic pot filled with a mixture of two types of commercial soil (without organic matter and poor in nutrients) prepared at the rate of 1:1. The soils were well-weathered volcanic ash Akadama and well-weathered pumice Kanuma. The pH of the mixture was 5.92 ± 0.02; the reader may refer to Agathokleous et al. (2016a) for more details about the soils. One-third of the pots of each EDU treatment were placed on a fully randomized design (pot-to-pot distance = 30 cm) in each of three ambient plots at the experimental nursery of Field Science Center of Hokkaido University. The plot-to-plot distance was > 30–100 m (the plots were different in each growing season). Throughout the growing seasons, the pots position was re-adjusted randomly biweekly to avoid edge effects. Likewise, the pots of each plot were subjected to rotation across plots on a monthly basis. Initial measurements of the saplings were taken as described for experiment I (“Experiment I” section). The average values of shoot height, crown span, stem diameter, and number of branches per sapling were 15.7 ± 0.11 cm, 6.8 ± 0.05 cm, 2.7 ± 0.02 mm, and 8.0 ± 0.13, respectively.

#### EDU treatments

The EDU treatments were 0, 200 and 400 mg EDU L^−1^ (200 mL per pot). EDU treatments were assigned randomly to saplings before the first application in the first growing season, and, then, each sapling was always treated with the same EDU treatment every 9 days during each growing season. Seven, eight, and eight saplings were allocated to EDU0, EDU200, and EDU400 treatments, respectively. The first EDU application was conducted on 29 July in 2014, on 14 April in 2015, on 24 April in 2016, and on 9 June in 2017. A total of 12, 22, 17, 13 applications were carried out in 2014, 2015, 2016, and 2017, respectively. Therefore, EDU had been applied 64 times throughout the 4 growing seasons, for a total of 12.8 L water solution per plant. EDU methodology was same with the one described for experiment I (“EDU treatments” section).

#### O_3_ treatments

Plants were grown under ambient O_3_ throughout the 4 growing seasons, in the open-field plots explained earlier (“Plant material preparation” section). Exposing plants to only ambient O_3_ (with no other O_3_ treatment) is a common practice in the research of EDU effects on plants (Oksanen et al. 2013; Carriero et al. 2015; Yuan et al. 2015; Fatima et al. 2019; Pandey et al. 2019). Because the ambient O_3_ level in Sapporo is relatively low compared to southern areas of the Northern Hemisphere, the plants were subjected to elevated O_3_ in the third (23 May to 1 October, 2016) and fourth (18 June to 1 October, 2017) growing seasons. The exposure to elevated O_3_ was conducted in the same FACE system with experiment 1 (“O_3_ treatments” section). In each of the 2016 and 2017 growing seasons, 2–3 saplings per EDU treatment were allocated to each of 3 FACE plots with elevated O_3_-Pots were rotated across FACE plots with elevated O_3_ on a monthly basis. Similar equivalent saplings non-used for research were placed in the ambient FACE plots to not affect the research with the vegetation growing in the plots. The average daily 10-h (08:00–18:00 Japan Standard Time, JST) O_3_ concentration was 22.3 ± 3.3 (s.d.), 34.3 ± 5.5, 53.2 ± 8.8, and 57.5 ± 11.5 nmol mol^−1^ during the growing seasons of 2014, 2015, 2016 and 2017, respectively.

#### Data collection

At the end of the experiment, growth and biomass were measured in all plants, as described for experiment I (“Experiment I” section). Stem diameter, shoot height and crown span were measured 6 times (November, 2014; June, July, August, and October, 2015; and May 2016), as described in “Experiment I” section.

### Data analysis

An alpha level of 0.05 was selected a priori for the statistical significance. Data were averaged per plant community (pot per EDU treatment) per FACE plot, so to provide 3 real replicates for each combination of EDU and O_3_ treatments. Data of experiments I and II were subjected to a Box-Cox transformation (Box and Cox 1964), as described by Agathokleous et al. (2016a).

Statistical hypothesis testing of experiment I data was done with Overall and Spiegel Method I Sum of Squares-adjusted (Howell and McConaughy 1982) General Linear Models (GLM) with treatment as fixed factors, randomized by FACE plot. Correlation between the total dry mass per plant community and the seedling survival in experiment I was tested by a simple linear regression analysis.

Biomass data of experiment II were tested with contrasts because of the a priori nature of the dose–response test design. Two degrees of freedom were partitioned into two comparisons: (1) EDU0 versus (EDU200 + EDU400), and (2) EDU200 versus EDU400. The former comparison (1) tests whether EDU treatment affected plants, while the latter comparison (2) tests whether the EDU effect differed between EDU200 and EDU400 treatments.

Growth data of experiment II were tested with repeated measures GLM, with treatment as fixed factor and time as within-subjects factor with 6 levels.

For significant treatment main effect regarding experiment I data sets and growth data of experiment II, Bonferroni post hoc test was applied for multiple comparisons among the experimental groups.

Data processing and statistics were performed with EXCEL 2010 (Microsoft, Redmond, CA, USA) and STATISTICA v.10 (StatSoft, Tulsa, OK, USA).

## Results

### Experiment I

#### Plant survival

Because of the susceptibility at the early ontogenic stage when the treatments began, some of the seedlings did not survive and died at some stage of the experiment, mostly early in O_3_ exposure (within 2 weeks from the exposure initiation). Hence seedling survival was calculated as the percentage of alive plants from the total number of plants per pot at the end of the experiment.

**Fig. 1:**
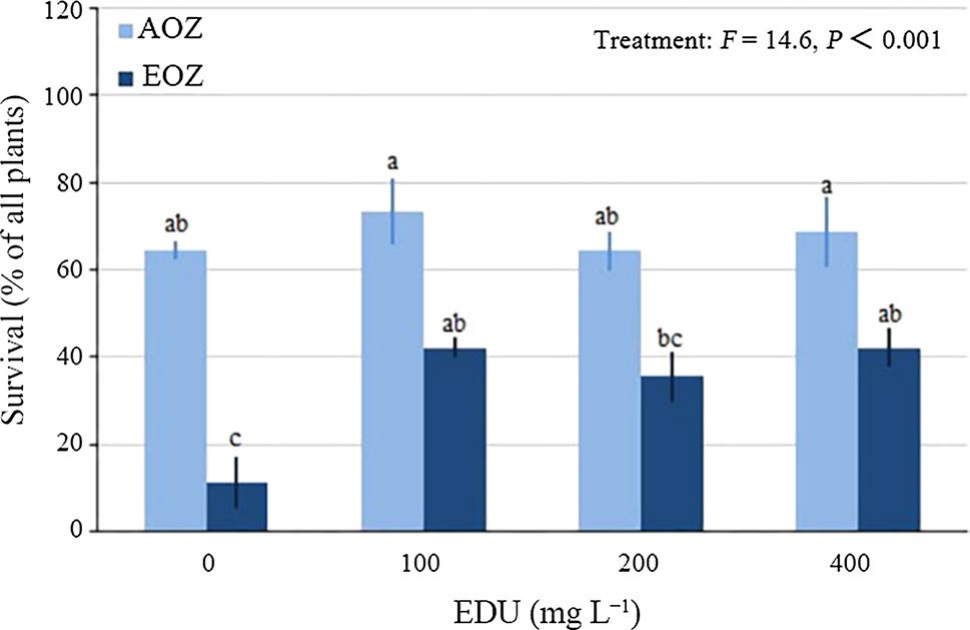
Survival of Japanese larch seedlings treated with various concentrations of ethylenediurea (EDU) and exposed to ambient (AOZ) or elevated O_3_ (EOZ) for about 3 months (experiment I). Different letters above the se bars indicate significant differences among different groups. Data are communities mean values ± se (*n* = 3). Data were tested with general linear model at *α *= 0.05. For significant main effect of Treatment, Bonferroni post hoc test applied for multiple comparisons

Treatment was a significant factor for plant survival (Fig. 1). Seedling survival of EDU0 group was 53% lower in EOZ than in AOZ. EDU100, EDU200 and EDU400 did not affect seedling survival relative to EDU0 in AOZ. However, EDU100, EDU200 and EDU400 increased seedling survival 31, 24 and 31%, respectively, over EDU0 group in EOZ. Although the difference between EDU200 and EDU0 in EOZ is not statistically significant, seedling survival between EDU200 in AOZ and EDU200 in EOZ is also not statistically significant, suggesting EDU200 also protected seedlings from mortality in EOZ.

#### Plant growth

**Fig. 2:**
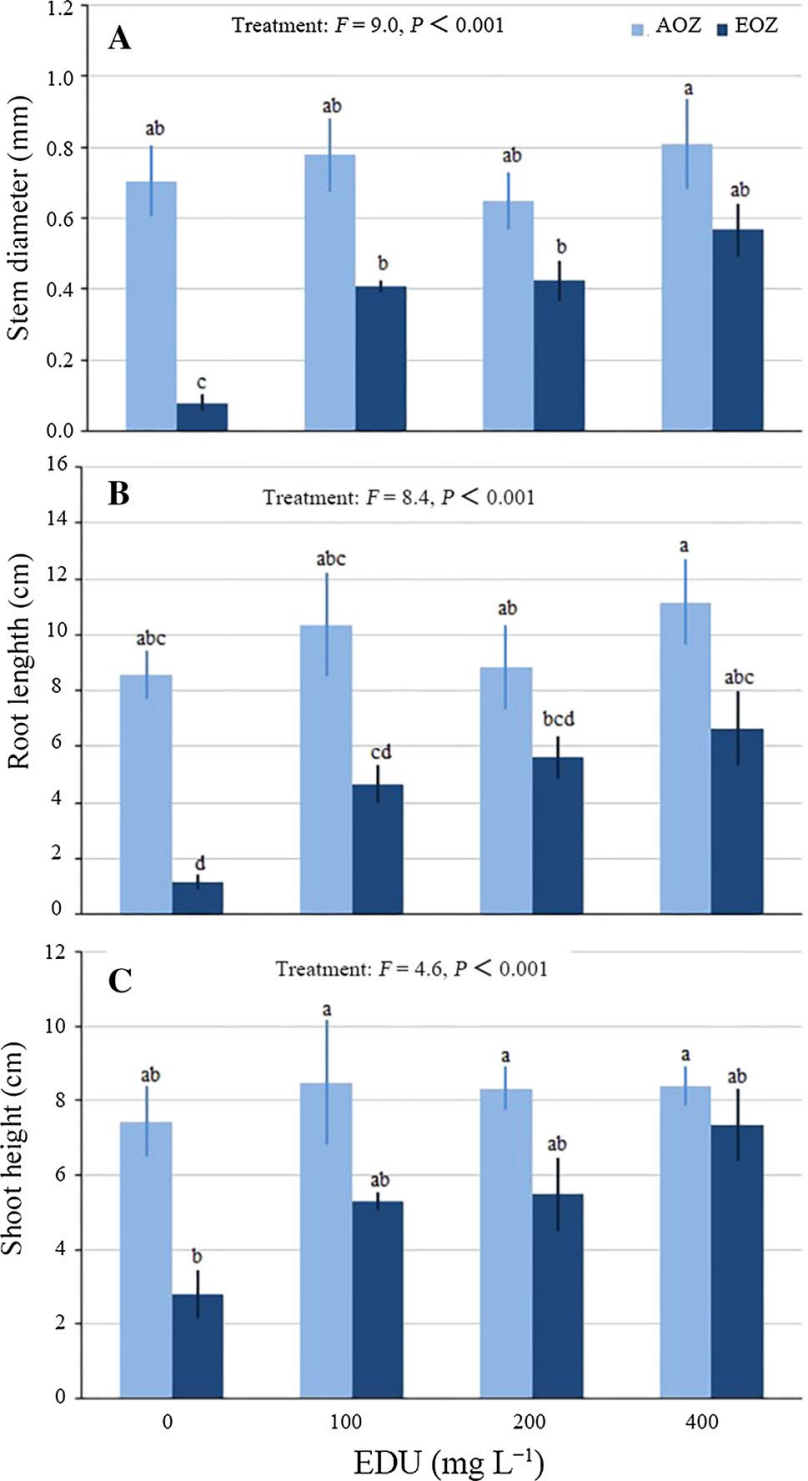
Growth of Japanese larch communities treated with various concentrations of ethylenediurea (EDU) and exposed to ambient (AOZ) or elevated O_3_ (EOZ) for about 3 months (experiment I). Different letters above the se bars indicate significant differences among different groups. Data are communities mean values ± se (*n* = 3). Data were tested with general linear model at *α *= 0.05. For significant main effect of Treatment, Bonferroni post hoc test applied for multiple comparisons

Average stem diameter (Fig. 2A) and root length (Fig. 2B) per plant community were significantly inhibited by EOZ in EDU0 (AOZ-EDU0 vs. EOZ-EDU0). However, EOZ did not significantly inhibit average stem diameter and root length per plant community in EDU100, EDU200 and EDU400, indicating that the three EDU treatments protected plant communities against EOZ-induced growth inhibition. Average root length of plant communities treated with EDU100 and EDU200 was not significantly different from average root length of plant communities treated with EDU0 in EOZ, indicating that EDU100 and EDU200, in contrast to EDU400, did not fully protect root length against EOZ-induced inhibition. No significant differences in average shoot height per plant community were observed among EDU treatments in either AOZ or EOZ and between pairs of same EDU treatment in AOZ and EOZ; a high variance existed (Fig. 2C).

**Fig. 3:**
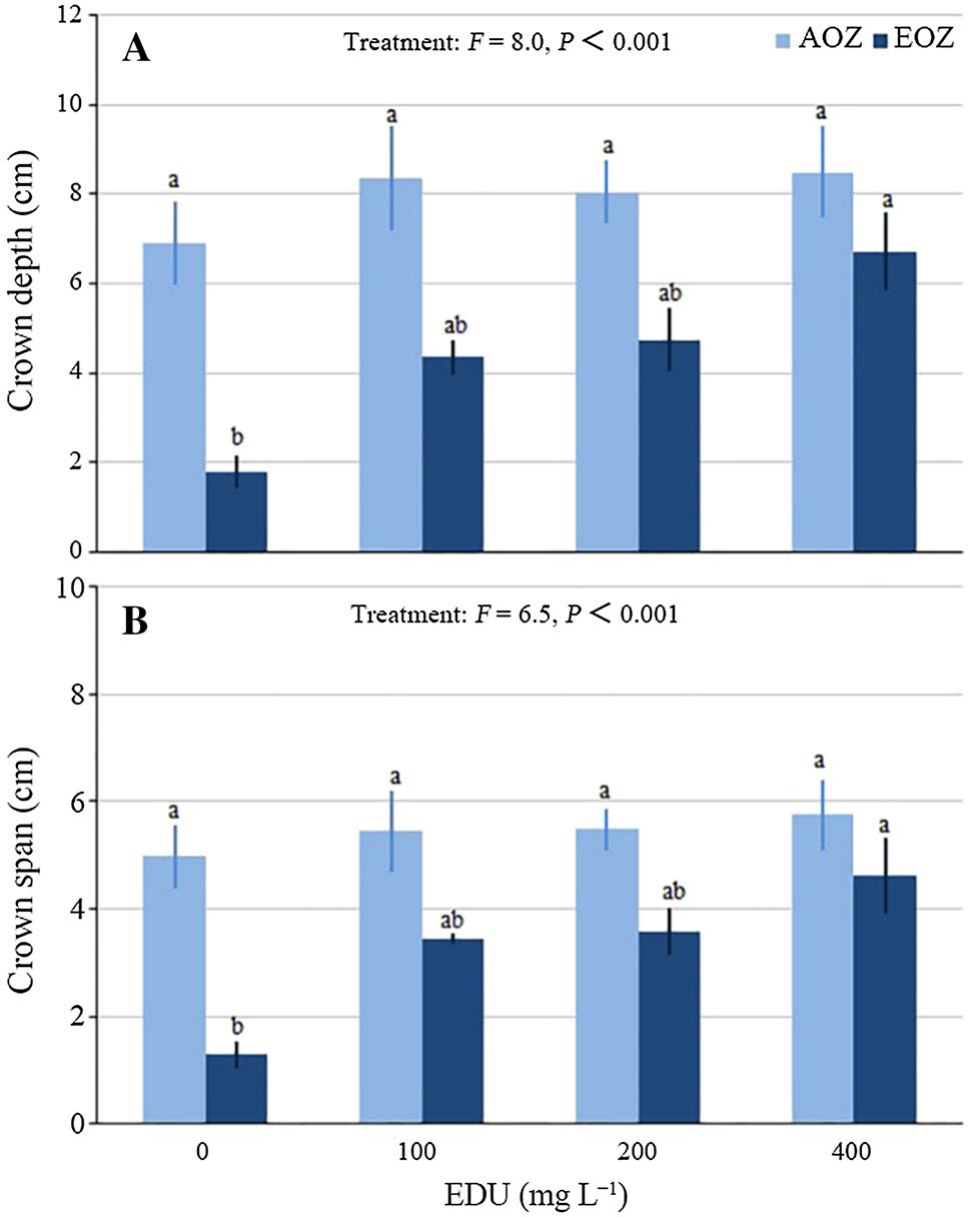
Crown growth of Japanese larch communities treated with various concentrations of ethylenediurea (EDU) and exposed to ambient (AOZ) or elevated O_3_ (EOZ) for about 3 months (experiment I). Different letters above the se bars indicate significant differences among different groups. Data are communities mean values ± se (*n* = 3). Data were tested with general linear model at *α *= 0.05. For significant main effect of Treatment, Bonferroni post hoc test applied for multiple comparisons

Average crown depth (Fig. 3A) and crown span (Fig. 3B) per plant community were significantly inhibited by EOZ in EDU0 (AOZ-EDU0 vs. EOZ-EDU0), but this was not the case in EDU100, EDU200 and EDU400 communities when compared with the respective EDU treatments between AOZ and EOZ. The EDU protection was full in EDU400 seedlings as (1) the means were similar between AOZ and EOZ for EDU400 and (2) EDU400 communities had significantly higher means than EDU0 communities in EOZ. However, EDU100 and EDU200 did not fully protect plant communities against EOZ-induced inhibition because the EDU100 and EDU200 communities had no significantly higher means than EDU0 communities in EOZ.

#### Plant biomass

**Fig. 4:**
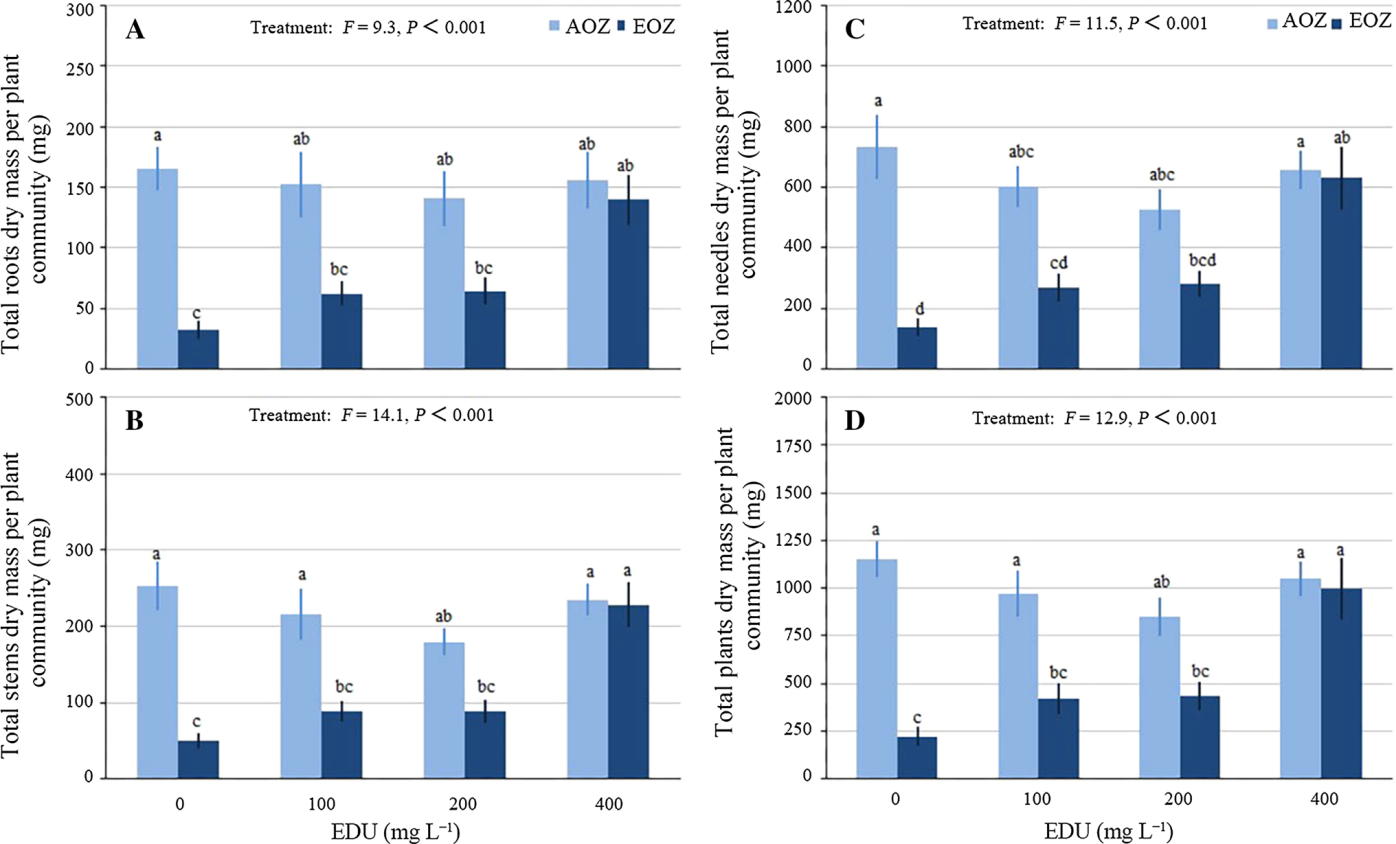
Biomass of Japanese larch communities treated with various concentrations of ethylenediurea (EDU) and exposed to ambient (AOZ) or elevated O_3_ (EOZ) for about 3 months (experiment I). Different letters above the se bars indicate significant differences among different groups. Data are communities mean values ± se (*n* = 3). Data were tested with general linear model at *α *= 0.05. For significant main effect of Treatment, Bonferroni post hoc test applied for multiple comparisons

Total roots (Fig. 4A), stems (Fig. 4B) needles (Fig. 4C) and plants (Fig. 4D) dry mass per plant community displayed the same response pattern: EDU100 and EDU200 did not fully protect seedlings against EOZ-induced inhibition, as EDU100 and EDU200 seedlings had no significantly higher mean values than EDU0 seedlings in EOZ; however, EDU400 fully protected seedlings against EOZ-induced inhibition. The total dry mass per plant community was positively correlated with the seedling survival (*y* = 13.275*x* + 93.944; *r* = 0.628; *F* = 14.3, *P* < 0.01).

For all plant traits assessed, EDU100, EDU200 and EDU400 treatments did not have a significant effect on seedlings in AOZ, indicating no significant effect of AOZ on seedling growth and biomass.

### Experiment II

#### Plant growth

Stem diameter, shoot height and crown span (data not shown) were significantly affected by time only (Table 1).

**Table 1:** Statistical results of growth of Japanese larch saplings treated with 0 (EDU0), 200 (EDU200) or 400 (EDU400) mg L^−1^ ethylenediurea (EDU) and exposed to O_3_ for 4 growing season (experiment II)

Growth trait	GLM results		
	Treatment	Time	Treatment × time
Stem diameter	*F* = 0.10, *P* = 0.904	*F* = 24.65, *P* < 0.001	*F* = 0.53, *P* = 0.865
Shoot height	*F* = 0.27, *P* = 0.767	*F* = 15.56, *P* < 0.001	*F* = 0.41, *P* = 0.940
Crown span	*F* = 0.57, *P* = 0.577	*F* = 34.12, *P* < 0.001	*F* = 0.90, *P* = 0.533

Growth was measured 6 times over time. Data were tested with repeated measures general linear model (GLM) at *α *= 0.05

#### Plant biomass

**Fig. 5:**
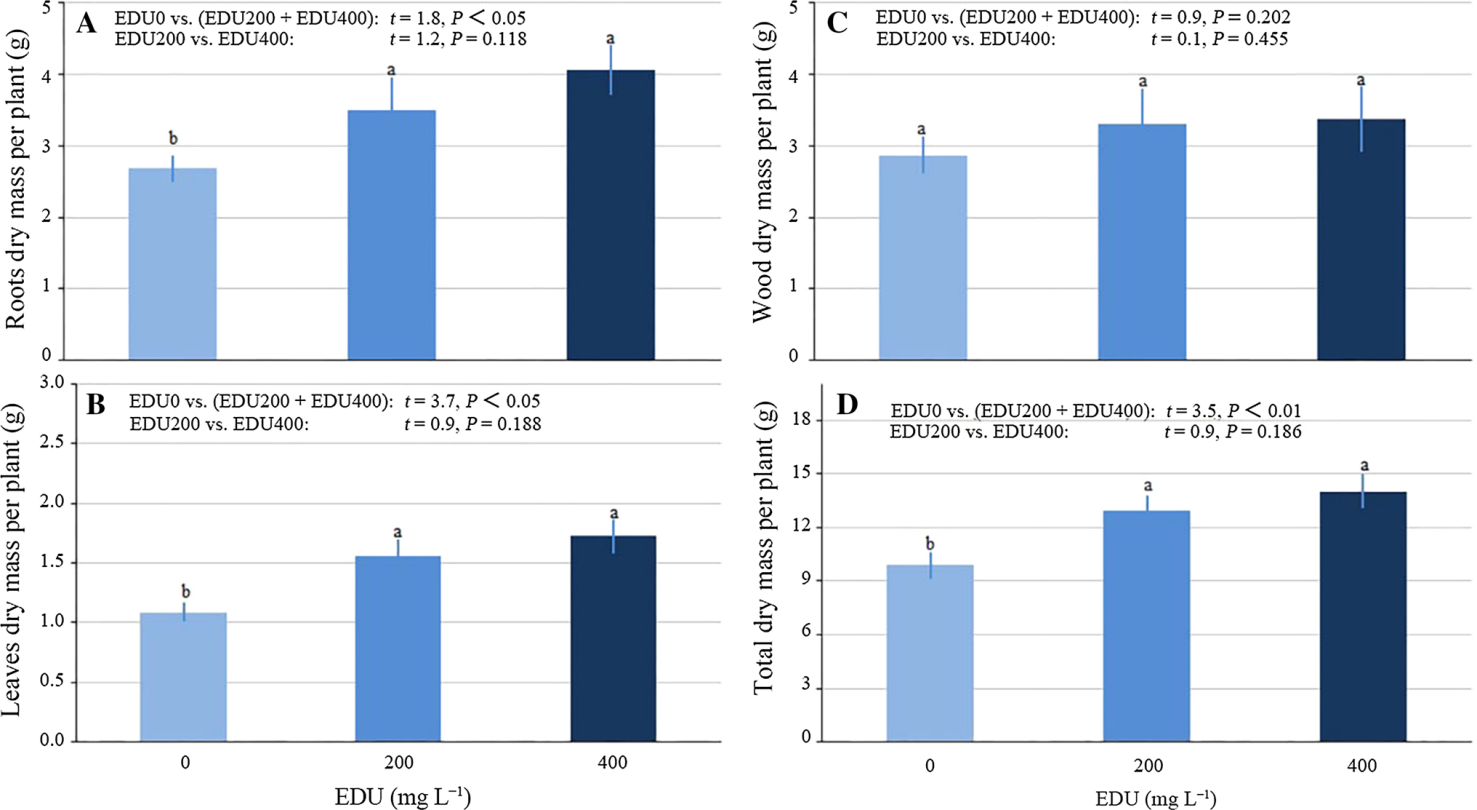
Biomass of Japanese larch saplings treated with 0 (EDU0), 200 (EDU200) or 400 (EDU400) mg L^−1^ ethylenediurea (EDU) and exposed to O_3_ for 4 growing season (experiment II). Wood dry mass is the sum of branches and stem. Different letters above the se bars indicate significant differences among different groups. Data are mean values ± se (*n* = 7–8). Data were tested with two contrasts at *α *= 0.05

EDU treatment did not significantly affect woody dry mass per plant (Fig. 5C). However, EDU treatment (EDU200 + EDU400) increased roots (Fig. 5A), leaves (Fig. 5B), and total (Fig. 5D) dry mass per plant by 40.8, 51.4, and 36.4% on average, respectively. There was no significant difference between the effect of EDU200 and the effect of EDU400. The ratio of root biomass to shoot biomass (R/S) was not affected by EDU (EDU200 + EDU400) (*t* = 0.74, *P* = 0.468), and there was no difference between EDU200 and EDU400 (*t* = 0.11, *P* = 0.910) (data not shown).

## Discussion

Experiments I and II documented adverse effects of elevated O_3_ on plant productivity (and plant size for experiment I). However, experiment I shows that ambient O_3_ did not affect Japanese larch communities with high intraspecific competition. This finding is in agreement with previous experiments with Japanese larch saplings, grown in the presence or absence of competition, within open-top chambers in the same area (Koike et al. 2012; Sugai et al. 2018). The effects of  high O_3_ concentrations on survival and biomass of generating communities grown in the presence of high intraspecific competition (experiment I) are more adverse than those found in previous experiments with saplings and tall trees of Japanese larch conducted at the same area (Koike et al. 2012; Wang et al. 2015; Agathokleous et al. 2017; Sugai et al. 2018) (see also experiment II), suggesting that regenerating seedlings and communities of Japanese larch may be more susceptible to elevated O_3_ than what experiments with individual saplings suggest. Collectively, it appears that communities of young generating seedlings of Japanese larch are susceptible to elevated O_3_ levels but not susceptible to current ambient O_3_ levels.

In a 6 year-long experiment with EDU applied to poplars in a Mediterranean climate with relatively high exposures of O_3_, O_3_ did not change the R/S ratio, suggesting that the reduced allocation to roots often found in short-term experiments may not reflect a long-term phenomenon (Carriero et al. 2015). Experiment II with Japanese larch saplings supports the finding and suggestion of Carriero et al. (2015).

EDU100, EDU200 and EDU400 treatments enhanced Japanese larch seedlings growth and survival in EOZ, and, thus, also enhanced the productivity of communities (in terms of dry matter) in experiment I. This enhancement appeared to be insufficient for EDU100 and EDU200, for most traits studied, while EDU400 offered the highest protection, indicating that the maximum stimulatory response to EDU occurred at 400 mg L^−1^. The concentration of 100 mg L^−1^ is lower than the overall range (150–450 mg L^−1^) reported for many species and genotypes (Agathokleous 2017), suggesting that concentrations below 150 mg L^−1^ should be considered when conducting cost–benefit evaluations for potential forestry applications.

Experiment II confirmed the protection by EDU200 and EDU400 found in experiment I, and provided evidence for long-term protection of Japanese larch against O_3_ damage. The finding of successful protection of this coniferous tree over multiple growing seasons is in agreement with the only long-term experiment studying EDU effects on plants (Hoshika et al. 2013; Katanić et al. 2014; Carriero et al. 2015; Giovannelli et al. 2019). In that experiment, weekly applications of 450 mg EDU L^−1^ protected the growth and productivity of a broadleaved hybrid poplar over multiple years, improved its seasonal sap flow by increasing leaf area for sapwood unit, and positively affected the community of mycorrhizal fungi (Katanić et al. 2014; Carriero et al. 2015; Giovannelli et al. 2019). Conversely to experiment I, where EDU200 did not provide as sufficient protection as EDU400, EDU200 offered a similarly sufficient protection with EDU400 in experiment II. This may be attributed to the fact that saplings were individually grown in experiment II, in the absence of competition for resources, and, thus, under lower level of stress than seedlings in experiment I. It can be postulated that the difference in EDU concentration needed to protect plants was not due to dilution effect attributable to plant size because EDU200 did not protect plants in experiment I (smaller plants − current-year seedlings; total foliage biomass per control community = 0.73 g) but protected plants in experiment II (larger plants − saplings; foliage biomass per control sapling = 1.09 g). The results of the present study, via two independent experiments and multiple EDU concentrations, illustrate that EDU does not affect plant growth and biomass linearly at concentrations smaller than the typical toxicological threshold. Such non-linear effects of EDU might be related to the mechanism of EDU protection against O_3_-induced damage, encouraging further studies where the effect of multiple EDU concentrations on growth and biomass would be linked to underpinning molecular mechanisms.

Preliminary evidence from broadleaved plant species suggests that the maximum amount of EDU needed is ≈ 10–30 mg m^−2^ leaf area per treatment (Agathokleous 2017). Leaf area was not measured in this coniferous species. Based on the foliage biomass measured at the end of the experiment in the control EDU0 treatments (to represent the maximum amount of EDU needed), the amount of EDU in each EDU200 treatment was 54.7 mg g^−1^ foliage (109.4 mg for EDU400) in experiment I and 39.6 mg g^−1^ foliage (79.3 mg for EDU400) in experiment II. Therefore, a maximum amount of 109.4 mg EDU g^−1^ foliage was sufficient to protect the seedling communities throughout the growing season in experiment I, whereas a maximum amount of 39.6 mg EDU g^−1^ foliage was sufficient to protect the individual saplings throughout the growing seasons in experiment II. Measuring the foliage biomass (as an alternative of area) in conifers over time is practically difficult because destructive sampling would require a considerably higher number of plants, but further evaluations of the amount of EDU per foliage biomass at different time points would be needed. Nevertheless, the present estimates can be helpful to calculate the required amount of EDU in Japanese larch plants with greater foliage.

## Conclusions

In conclusion, EDU concentrations in the range of 200–400 mg L^−1^ could protect Japanese larch generating communities of seedlings and individual saplings against O_3_ damage. However, the protection of concentrations ≤ 200 mg EDU L^−1^ was only partial when seedlings were coping with high competition and a considerably high level of O_3_ stress, thus, 400 mg EDU L^−1^ would be needed for plants under high competition and stress. This study also provides evidence that EDU can be also used for active or passive O_3_ biomonitoring of generating communities in Japanese larch forests, where O_3_ is not measured by technological instruments. Hence, the results of this study are important for both protecting Japanese larch against O_3_ damage and monitoring O_3_ for potential risks to Japanese larch plantations.

Further studies are needed to identify the optimum amount of EDU needed for sufficient protection. However, this is a challenging task for conifers, as the amount of EDU needed should be calculated as a function of the foliage area or mass. Nevertheless, studies associating EDU amount, foliage, and growth traits may permit the calculation of EDU amount based on simple plant size traits in the future.

## Electronic supplementary material

Below is the link to the electronic supplementary material.

Supplementary material 1 (PDF 533 kb)
