# Supplementary material for: Ethylenediurea (EDU) effects on Japanese larch: an one growing season experiment with simulated regenerating communities and a four growing season application to individual saplings
Source: J For Res (Harbin). 2020 Sep 30;32(5):2047–57. doi: 10.1007/s11676-020-01223-6 (PMC7525765; doi:10.1007/s11676-020-01223-6)
Supplement: Supplementary file 1 — Supplementary material 1 (PDF 533 kb) [file 11676_2020_1223_MOESM1_ESM.pdf]

Supplementary Information

Ethylenediurea (EDU) effects on Japanese larch: an one growing season experiment with simulated regenerating communities and a four growing season application to individual saplings

Evgenios Agathokleous[1,2,3*], Mitsutoshi Kitao[3], Xiaona Wang[2,4], Qiaozhi Mao[2,5], Hisanori Harayama[3], William J Manning[6], Takayoshi Koike[2,7, 8]

[1] Key Laboratory of Agrometeorology of Jiangsu Province, Institute of Applied Ecology, Nanjing University of Information Science and Technology, Nanjing 210044, Jiangsu, China;
[2] Division of Environment and Resources Research, Research Faculty of Agriculture, Hokkaido University, Sapporo 060-8589, Hokkaido, Japan;
[3] Hokkaido Research Center, Forestry and Forest Products Research Institute, Sapporo 062-8516, Japan;
[4] College of Landscape Architecture and Tourism, Hebei Agricultural University, No.2596 Lekai South Street, Lianchi District, Baoding 071000, China;
[5] College of Resources and Environment, Southwest University, Chongqing 400700, China;
[6] Department of Plant, Soil and Insect Sciences, University of Massachusetts, Amherst, MA, USA;
[7] Shenzhen Graduate School of Environment & Energy, Peking University, Shenzhen 518055; China;
[8] Research Center for Eco-Environmental Science, Chinese Academy of Science, Beijing 100085, China.
[1]Corresponding author. E-mail address: evgenios@nuist.edu.cn (E.A.) ORCID ID: 0000-0002-0058-4857

The FACE (Free Air Controlled Exposure) system for exposing communities of deciduous plant species to O_3 in northeast Asia

1. BACKGROUND

Global change forced scientists to study effects of changing environment on vegetation (e.g. Matyssek et al. 2013). In the need to investigate tropospheric carbon dioxide (CO_2) and near-surface ozone (O_3) effects on plants, several engineering design approaches have been developed (Kobayashi 2015). The first approach is by using closed systems, which are different kinds of closed facilities restricting interactions with the natural environment (Menser and Heggestad 1964; Hill 1967; Berry 1970; Rafarel and Ashenden 1991). The second approach is by utilizing semi-open systems, which are more realistic than closed chambers; the most commonly used method of semi-open systems is open-top chambers (OTCs, Barbee et al. 1973; D'Andrea and Rinaldi 2010). An important shortcoming of the first and second approaches is the isolation of plants from natural environment where many uncontrolled biotic (e.g. insects) and abiotic factors (e.g. temperature, moisture, light, wind speed) influence or interact with the plants.

Notably, plants are often grown in pots where the root system is restricted, imbedding, thus, authentication of the findings with what would be observed under unrestricted environment. In order to overcome such shortcomings, the third approach, open air systems, has been developed. This approach includes mainly Free Air Controlled Exposure (FACE) systems (Werner and Fabian 2002; Karnosky et al. 2008; Norby and Zak 2011; Tang et al. 2011; Matyssek et al. 2013; Koike et al. 2015 for a list with information on the global CO_2 FACEs) and, more rarely though, localized exposure systems with tubes wrapped around tree branches (Velikova et al. 2005). It should be noted that all methods have advantages and

disadvantages (Unsworth et al. 1984; Macháčová 2010); for example, O_3-FACE systems require much higher financial support.

O_3-FACE is a method used by plant biologists, ecologists and ecophysiologists to enrich air with O_3 in a particular experimental area and allow the response of plant growth to be measured without restricting root growth and in interaction with the natural environment, in contrast to closed or semi-open systems. Severe negative effects of elevated mixing ratios of O_3 on vegetation urged dozens of scientists to engage O_3 research with annual or perennial plants (Agathokleous et al. 2015a, 2015b, 2016a; Ainsworth et al. 2012; Koike et al. 2013; Harmens et al. 2015; Chappelka and Grulke 2016; Sicard et al. 2016; Wang et al. 2016). However, opposed to the development of numerous CO_2 FACE systems around the globe (Koike et al. 2015), only few O_3 FACE systems for trees, with replicated experimental unit, have been operated –the most research has been carried out in closed or semi-open facilities (Wulff et al. 1992; Oksanen 2001; Nunn et al. 2002; Werner et al. 2002; Karnosky et al. 2003; Matyssek et al. 2013; Kitao et al. 2015; Paoletti et al. 2017).

O_3 impacts into vegetation are influenced by soil fertility (Agathokleous et al. 2016a; Shi et al. 2017). Thus, the influence of soil fertility and plant mineral status should be critically studied along with O_3. For example, a large proportion of global potential arable soils are infertile in phosphorus, while severe phosphorus scarcity is expected in future (Zheng 2010; Cordell et al. 2011; Cordell and Neset 2014; Reijnders 2014). Despite the importance of soil, and obviously for technical difficulties, there is not hitherto an O_3-FACE system for trees that employs soil as additional factor. Meanwhile, O_3 effects on plant may vary with growing substrate types, and fertility and properties of soil (Agathokleous et al. 2016a), something that is particularly important during the early stages of forest succession (Bazzaz 1996).

An O_3 FACE system that would allow the investigation of the effects of elevated O_3 on trees growing as a community in different kinds of soil was developed. This system was designed to simulate the early stages of the forest succession and thus is of particular importance to forest ecology research.

2. FACE' DEVELOPMENT IN 2014-2015

2.1. Experimental area

The O_3-FACE system was established at Sapporo Experimental Forest of Hokkaido University, located at Sapporo, Japan (43°.04' N, 141°.20' E, 15 m a.s.l.), in the year 2014. This location falls in a transition zone from cool temperate to boreal forests which is part of the Asian boreo-nemoral ecotone with relative sensitivity to global climate change (Uemura 1992; Matsuda et al. 2002). The experimental forest stand is located in the campus of Hokkaido University, in a central area of Sapporo city.

2.2. Meteorological conditions

The snow-free period for 2014 and 2015 lasted from early May to mid-November. Air temperature, wind speed, relative humidity, sunshine duration and total precipitation (Table S1, see the bottom of this document) were monitored by a nearby station at Sapporo (WMO, ID: 47412, 43°03.6'N 141°19.7'E), which is operated by the Japan Meteorological Agency (2016).

2.3. Experimental design

The O_3 treatments were ambient (AOZ) and elevated (EOZ) O_3, with three site replicates for each treatment. Each, approximately circular, plot (diameter=6.5 m) of AOZ and EOZ was surrounded by a metallic structure which consisted of six vertical metallic pipes (height=5m). The vertical metallic pipes were interconnected with horizontal metallic pipes at 1-m vertical interval. The architectural design of the free-air O_3-enrichment plots and the metallic structure of each plot are illustrated in Fig S1 and S2, respectively.

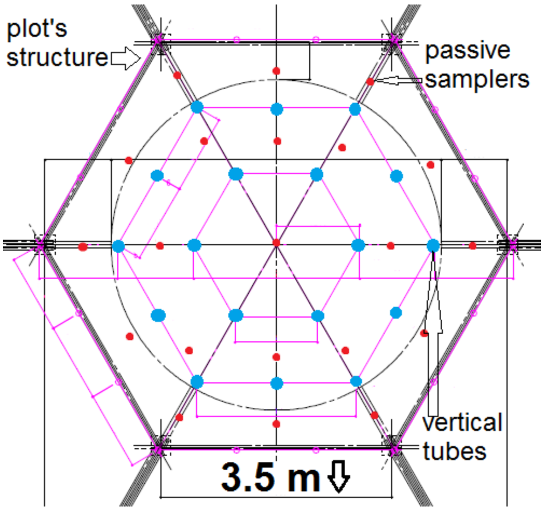

Fig S1. Architectural design of a free-air O_3-enrichment plot. The peripheral thicker lines (black in color version) represent the outer metallic scaffolding of the plot; around it a Teflon tube (at 0.5 and 1.5 m above ground level) was releasing O_3-enriched air in the plot. The fine lines (pink in color version), which form the two inner hexagons, represent the fixed nest on which the vertical Teflon tubes releasing O_3-enriched air (filled circles of larger size; blue in color version) were spatially arranged. Each vertical tube was connected with a horizontal tube, fixed on the nest, which reached the edge of the plot and then became vertical to connect with the buffer tank. The 21 smaller size filled circles (red in color version) indicate the position of the poles on which passive samplers were fixed at the heights of 1 and 1.5 m above ground level.

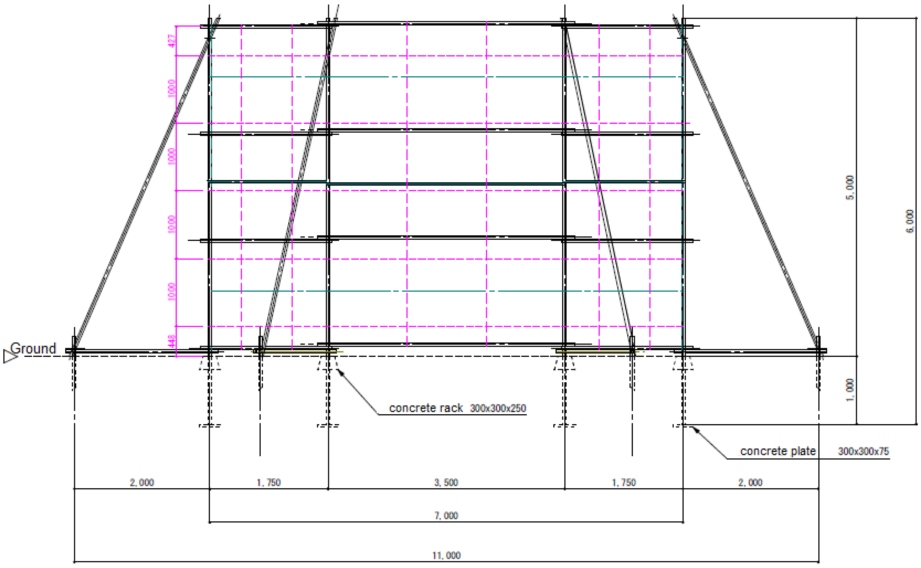

Fig S2. Metallic structure surrounding each experimental plot. The unit for the values shown in the figure is mm.

Within each O_3 plot, three types of soil were employed: a) brown forest soil (BF: Dystric Cambisols); b) BF mixed with immature volcanic ash plus pumice soil at a rate of 1:5 v/v (VA; Vitric Andosols); and c) BF mixed with serpentine soil at a rate of 1:5 v/v. BF and VA parts covered an area of approximately 42 % each while serpentine soil covered an area of approximately 16 % (Fig S3). Since BF is native to the experimental plots, soil in the surface 30 cm layer was removed from each soil site. For BF, the same soil was stirred and placed back. For VA and serpentine, the removed soil was mixed with volcanic ash or serpentine soil which was brought from Tomakomai Experimental Forest of Hokkaido University (Kim et al. 2011) and the eastern part of Teshio Experimental Forest (Watanabe et al. 2012), respectively, where they are native. BF is typical type of soil for deciduous broad-leaved forests which is fertile enough to support efficient plant growth (Kayama et al. 2011, Kim et al. 2011).

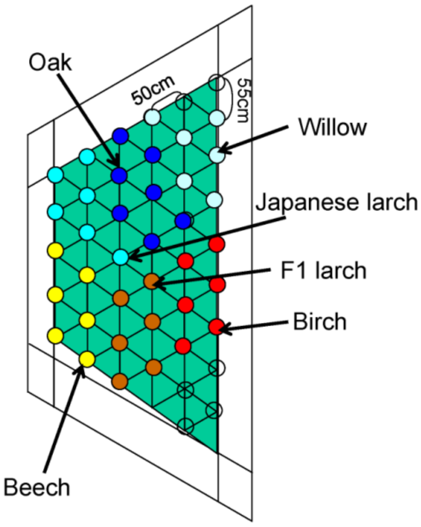

Fig. S3 Spatial arrangement of the soils and tree species in the experimental plots. The soil represents brown forest soil (BF; Dystric Cambisols)) or BF mixed with immature volcanic ash plus immature volcanic ash soil (VA; Vitric Andosols) which both have the same area. If this is BF, then VA is at the opposite site (right hand). A smaller area existed at the south (bottom of the illustration) which represented brown forest soil mixed with serpentine soil (serpentine). Serpentine differs from BF and VA in that there is no willow and only four individuals are planted per tree species. To separate run-off of serpentine to BF and VA, a plastic sheet was implanted into the soil, at a depth of 30 cm. The 2yr-old tree seedlings planted were Siebold's beech (*Fagus crenata* Blume), Japanese white birch (*Betula platyphylla* var. *japonica*), Mizunara oak (*Quercus mongolica* var. *crispula*), willow (*Salix udensis* Trautv. & C.A.Mey.), Japanese larch (*Larix kaempferi* (Lamb.) Carr.) and hybrid larch F_1 (*Larix gmelinii* var. *japonica* (Maxim. ex Regel) Pilg. × *L. kaempferi*). The length of one of the sides of the plot is shown (3.5 m, line is indicated by an arrow) to permit the calculation of any real dimensions if needed.

Volcanic ash soils are acidic, P deficient and N poor (Schmincke 2004; Kayama et al. 2011; Kam et al. 2015). Serpentine soils (derived from serpentine rock) have high pH and are characterized by high content in metals, such as Mg, that are harmful to plants and low content in essential elements for plants, such as Ca (Brady et al. 2005; Kayama et al. 2006; Kayama and Koike 2015). Earlier, for a decadal period of time (until 2013), these plots had been used for a free-air CO_2-enrichment (FACE) system (Koike et al. 2015; Agathokleous et al. 2016c). The plots were established on 13th May 2014.

On the same day (after the preparation of the plots and before the plantation), the pH of the soils was measured using a pH-meter (B-712, HORIBA, Kyoto, Japan). Three pH measurements were taken from the water used. Then, twelve samples of 50 g each prepared for each soil (i.e. 36 samples in total), from six FACE plots (two samples per FACE). Five soil samples of 10 g each was taken randomly from each plot, and were combined into 50 g of soil samples. The pH was measured for a mixture of 1:2 soil and water. Each sample was stirred for 30 sec every 5 min, for 30 min, and then left for some times to stabilize before the measurement.

On 15th May 2014, seedlings of five species were planted in the three soils: (a) Siebold's beech (*Fagus crenata* Blume); (b) Japanese white birch (*Betula platyphylla* var. *japonica*); (c) Mizunara oak (*Quercus mongolica* var. *crispula*); (d) Japanese larch (*Larix kaempferi* (Lamb.) Carr.); and (e) hybrid larch F_1 (*Larix gmelinii* var. *japonica* (Maxim. ex Regel) Pilg. × *L. kaempferi*). In mid. June, cuttings of willow (*Salix udensis* Trautv. & C.A.Mey.) were planted in BF and VA soils. Siebold's beech, Japanese white birch, Mizunara oak and the willow are deciduous broad-leaved species while the two larches are deciduous conifers. All the broad-leaved species are native to Hokkaido island, Japan. The spatial arrangement of soils and tree species in the plots are illustrated in Fig S3. Information on the properties of BF and VA soils can be found in Watanabe et al. (2013a) and Eguchi et al. (2005, 2008). Details on the soil properties of BF, VA and serpentine sites can be found in Shi et al. (2017). Overall, BF soil has lower Cr content than VA and serpentine soils; VA soil has higher Fe and Al content than BF and serpentine soils; serpentine soil has higher Mg, Ni, Cr and Mg/Ca ratio and lower K, Ca, Mn, Al and N content than BF and VA soils.

2.4. Ozone treatment

This FACE system employs the method of O_3 exposure used at Kranzberg Forest in Germany (Nunn et al. 2002; Werner and Fabian 2002). This system is also similar to the O_3 FACE towers established in the same experimental forest (Watanabe et al. 2013b), however the latter tower-type system was lacking replicated experimental units and its operation was discontinued in 2016. Recently, Paoletti et al. (2017) also reported an O_3 FACE system in Italy which implements this O_3 exposure methodology.

Ambient O_3 concentrations were monitored continuously by an O_3 monitor (Model 202, 2B Technologies, Boulder CO, USA) of which the measurement principle is based on UV Absorption at 254 nm. The O_3 monitor was located at a distance of approximately 20 m from the AOZ plots and was recording O_3 data at a 1 m interval.

The O_3 treatments were ambient O_3 (AOZ) and elevated O_3 (EOZ). The efficiency of the system was assessed in the first two years of its operation. Plants were exposed to EOZ from August 15th to October 26th in 2014 and from April 24th to October 26th in 2015, during the daytime. This system was initially designed to maintain EOZ at a target mixing ratio of 70 nmol mol^{-1}, about two times the ambient O_3 mixing ratio at the experimental area. However, the attainment capacity of this system may deviate importantly under stronger winds which commonly occur at the experimental area (Watanabe et al. 2013b). Therefore, to approximate the target, the target mixing ratio was set to 80 nmol mol^{-1}. When the target level is lower than the target one, opening of the three-way control valve increases to supply more O_3. When the target level exceeds 80 nmol mol^{-1}, the opening of the three-way control valve decreases (closing); then the O_3 level in the plot decreases.

Ozone generated from pure oxygen was diluted with pressurized ambient air and released into the rings by eighteen 85 % virgin Teflon tubes (length = 2.5 m, inner diameter = 6 mm) hanging down from a nest fixed above the plants (2.5 m above ground level), at fixed distances among them, and two Teflon tubes (length = 21 m, inner diameter = 6 mm) fixed horizontally around the plot at 0.5 and 1.5 m above ground level for each EOZ plot. However, the nest can be unfixed and fixed at a higher position, according to the plant height. Teflon is a known formula based on polytetrafluoroethylene (PTFE), a synthetic fluoropolymer of tetrafluoroethylene.

Each tube had one hole every 50 cm (i.e. one 2.5 m long tube had five holes), and an end-cap at the bottom. The end-cap had also a hole to avoid accumulation of humidity and maintaining pressure in the tubes. Each of the eighteen vertical tubes was connected at their top with a horizontal tube fixed on the nest (until the edge of the plot when it was again vertical) which was in turn connected to a buffer tank. All the holes had a diameter of 1.5 mm. The blowing amount of air in the plot was 1.53 L min^{-1}. The spatial arrangement of the tubes in the EOZ plots is schematically represented in Fig S1. The buffer tank was connected, with two Teflon tubes, to the mixing tanks in which the air containing O_3 was diluted with ambient air before passes into the buffer tank and thereby into the horizontal and vertical air-releasing tubes of the experimental plot. Each EOZ plot had its own control station in which all the instruments were established.

An SM70 Fixed Ozone Monitor (Aeroqual Ltd., Auckland, NZ) was installed in the center of each EOZ plot, in an SM70 enclosure (Aeroqual Ltd., Auckland, NZ) which is pre-drilled and fitted with a cable gland for facilitating a cable connection to the SM70 relay and data output connectors. The SM70 enclosure is placed on a metallic pipe so as the measuring height to not be fixed and can be adjusted to plant height requirements. Air is sampled, and the SM70 response time is one minute. Calibration is done with zero gas (no O_3) and 1000 nmol O_3 mol^{-1} (as a span gas). The signal of the O_3 sensor is used to control O_3 at the target level through a proportional–integral–derivative (PID) control loop feedback mechanism (controller) algorithm.

The O_3-enrichment control system was controlled with a Digital Indicating Controller SDC31 (Azbil Corporation, Tokyo, JP) which has an accuracy of ±0.2% FS and offers a standard PID control and an advanced neural/fuzzy PID. This PID performs process diagnostics and reduces overshoot. A Hioki LR5042-20 (HIOKI E.E. Corporation, Nagano, JP) recorded O_3 data on a two-minute interval. Data were wirelessly transferred and stored into a data logger (LR5092, HIOKI E.E. Corporation, Nagano, JP). A light sensor (EE4313, Panasonic Industrial Devices SUNX Co., Ltd., Aichi, JP) was installed outdoors the control room of each EOZ plot and connected with the control system in order to provide feedback on the hours of active O_3 enrichment. In a similar way, a wind speed sensor was installed on the top of the metallic structure of each EOZ plot and connected with a Digital Alarm Combined Wind Vane Anemometer (Isuzu Seisakusho Co. Ltd., Tokyo, JP) and the control system in order to regulate the opening/closing of the three-way control valve for O_3 supply. When the wind speed was higher than 5 m s^{-1}, the O_3-enrichment system stopped. In the worst scenario that O_3 concentration in the plot reached much higher levels than the target concentration, the maximum closing time of the control valve was 3 min. The O_3 control system of each EOZ plot is autonomous, meaning that there was one control system for each plot.

In order to check the efficiency of the O_3-enrichment system, 21 poles were installed in an EOZ ring (Fig S1), each of which had two Ogawa O_3 passive samplers (Ogawa & Co., Ltd, Kobe, JP): one at the height of 1 m (around the lowest part of the canopy) and the other at the height of 1.5 m (around the top of the canopy). Each passive sampler was setup in an opaque plastic shelter (height = 14.5 cm, radius = 4 cm) which was further covered with aluminum foil; all shelters had the same size. Passive sampling lasted for 15,650±41 (hereafter mean ±S.D.) min. The data of the passive sampling were converted to mixing ratio by using the following conversion equation:

$$O_3(nmol\,mol^{-1}) = \alpha_{O_3} \times W_{O_3}/t,$$

where α_{O_3} is the nmol mol^{-1} mixing ratio conversion coefficient (nmol mol^{-1} min ng^{-1}), W_{O_3} is the O_3 quantity (ng) converted from the NO_3 quantity collected and t is the exposure duration (min). The α_{O_3} was calculated using the equation:

$$\alpha_{O_3} = 46.2 \times 10^2 \times \left(293/(273+T)\right)^{1.83}/(9.94 \times ln(t) - 6.53),$$

where T is the ambient temperature in degree Centigrade and t is the exposure duration in minutes. The equations are shown in Takeda and Komatsu (2007).

2.5. Exposure

When measured with water of pH=6.57±0.10, BF, VA and serpentine soils had a pH of 5.69±0.35, 5.09±0.26 and 6.45±0.28, respectively.

The 10-hour (07:00-17:00) mean O_3 levels of AOZ treatment were 20.66±5.52 and 34.7±7.55 nmol mol^{-1} in 2014 and 2015, respectively; S.D. indicates hour-to-hour fluctuations across the season. Notably, plants in AOZ condition are exposed to practically zero AOT40 (index of accumulated exposure to O_3 over the threshold of 40 nmol mol^{-1}, Mills et al. 2007). The relatively low ambient O_3 levels in the experimental area permit fair comparisons between O_3 treatments, meaning that there is a big difference between AOZ and EOZ levels which results to big difference in the accumulated exposure of plants to O_3.

The 10-hour (07:00-17:00) mean O_3 levels in the three EOZ plots were respectively 65.76±9.83, 61.68±11.94 and 62.0±11.52 nmol mol^{-1} in 2014 and 71.46±9.39, 76.02±7.56 and 69.25±10.51 nmol mol^{-1} in 2015. For the same hours, the minimum O_3 levels in the three EOZ plots were 36.14, 33.21 and 33.33 nmol mol^{-1} in 2014 and 31.55, 49.22 and 30.08 nmol mol^{-1} in 2015. Similarly, the maximum O_3 levels in the three EOZ plots were 103.2, 100.9 and 86.4 nmol mol^{-1} in 2014 and 96.0, 106.9 and 90.1 nmol mol^{-1} in 2015. As a measure of the closeness of the actual O_3 levels to the targets, the target achievement ratio (TAR) was also calculated as the ratio of measured O_3 mixing ratio to target O_3 mixing ratio (Okada et al. 2001). TAR in the three EOZ plots was 0.82±0.12, 0.77±0.15 and 0.78±0.14 units in 2014 and 0.89±0.12, 0.95±0.09 and 0.87±0.13 units in 2015. The mean, minimum, and maximum O_3 levels and TAR were calculated for each plot using hourly means across the season; S.D. indicates hour-to-hour fluctuations across the season.

The mean daily O_3 mixing ratio measured by passive samplers in 2014 was 43.2±7.2 and 39.2±4.3 nmol mol^{-1} at the two different heights, averaging to 41.2±6.2 nmol mol^{-1}; this mixing ratio is very close to the 24-h mean of the EOZ plots (41.06±14.27 nmol mol^{-1}, n=6) when measured by the instrumental O_3 analyzers during the O_3 exposure periods of both

years. The relatively low standard error of the passive samples for each height indicates that the distribution of O_3 within plot was sufficiently regulated.

Currently, only three O_3 FACE systems for trees exist in the global. The first one, and oldest of the three, is the one we technically present here, operating since the spring of 2014. The second one is the free air O_3 exposure (FO_3X) case-study system of FACE, located at Sesto Fiorentino, near Florence, Italy (43°48′59″N, 11°12′01″E, 55 m a.s.l.), described by Paoletti et al. (2017). The FO_3X FACE employs a split-plot experimental design with nine plots 5×5×2 m; there are three lines of replicated O_3 treatments at a distance of 17 m among them (Paoletti et al. 2017). In this FACE system, individual plants have been hitherto grown in pots (Hoshika et al. 2017; Zhang et al. 2017). The third and newest one is the FACE system located in Yanqing district, Beijing, China (116.0°E,40.5°N), in an area of 4 ha, which includes a Denuder system for long-term atmospheric sampling (DELTA). This FACE system employs two O_3 levels, one at ambient O_3 concentration and another set at 1.5 times time ambient O_3 level. Each O_3 treatment has four plot replicates. The size of each plot is 16×16 m (Feng, personal communication). In this FACE system, communities of poplars are growing directly into the ground (no pot-grown).

The FACE system of Hokkaido University, Japan, is the first system in Asia (Koike et al. 2013) and the only currently exists in the global for exposing communities of tree saplings of different taxa to O_3-enriched atmosphere. This system provides the minimum influence of the facilities on the natural environment, thus permitting studies on the interactions with the natural environment, such as plants and insect herbivores. Still, this is the first O_3 FACE employing soil as an additional factor and this is of benefit. The location of this FACE system is a further advantage: The technology which is available nowadays does not permit FACE systems to reduce the ambient O_3 levels which are elevated in several parts of the world. In contrary to the other available FACE systems, this FACE system is located in an area with relatively low ambient O_3 levels and thus provides a more realistic comparison in the sense that plants in areas with already highly elevated O_3 levels may be under background stress.

Acknowledgements: The authors appreciate Prof. Rainer Matyssek of Technological University of Munich, Germany, and Dr. Elena Paoletti of the National Council of Research, Italy, for encouraging the development of this system. Authors gratefully thank Zhaozhong Feng of the Research Center for Eco-Environmental Sciences, Chinese Academy of Sciences, Beijing, P.R.China for providing information about their research facilities. Thanks are also due to Mr. Tatsushiro Ueda (Dalton Co. Ltd., Hokkaido) for designing and arranging the O_3-FACE system and continuously supporting its operation. They also acknowledge Dr. Takashi Yamaguchi of the Environmental Center, HRO, Sapporo, Japan, for his assistance in setting and analyzing the O_3 passive samplers. Evgenios Agathokleous was an International Research Fellow (ID No: P17102) of the Japan Society for the Promotion of Science (JSPS). Support was provided in part by JSPS and the Japan Science and Technology Agency (JST) (Grant No. JPMJSC18HB to TK via Prof. T. Watanabe).

References

Agathokleous E, Saitanis CJ, Koike T (2015a) Tropospheric O_3, the nightmare of wild plants: A review study. J Agr Meteorol 71: 142-152.

Agathokleous E, Koike T, Watanabe M, Hoshika Y, Saitanis CJ (2015b) Ethylene-di-urea (EDU), an effective phytoprotectant against O_3 deleterious effects and a valuable research tool. J Agr Meteorol 71: 185-195.

Agathokleous E, Saitanis CJ, Wang X, Watanabe M, Koike T (2016a) A review study on past 40 years of research on effects of tropospheric O_3 on belowground structure, functioning and processes of trees: a linkage with potential ecological implications. Wat Air Soil Poll 227: 33.

Agathokleous E, Watanabe M, Eguchi N, Nakaji T, Satoh F, Koike T (2016c) Root production of *Fagus crenata* Blume saplings grown in two soils and exposed to elevated CO_2 concentration: an 11-year free-air-CO_2 enrichment (FACE) experiment in northern Japan. Wat Air Soil Pollut 227: 187.

Ainsworth EA, Yendrek CR, Sitch S, Collins WJ, Emberson LD (2012) The effects of tropospheric ozone on net primary productivity and implications for climate change. Annu Rev Plant Biol 63: 637-61.

Barbee DG, Goplen SP, Thomas III OB, Nuckolls CE (1973) A Review categorizing engineering design techniques of plant environmental simulators. J Agric Eng Res 18: 13-29.

Bazzaz FA. 1996. Plants in changing environments: Linking physiological, population, and community ecology. Cambridge University Press, Cambridge. pp. 332.

Berry CR (1970) A plant fumigation chamber suitable for forestry studies. Phytopathology 60: 1613-1615.

Brady K, Kruckberg A, Bradshaw H (2005) Evolutionary ecology of plant adaptation to serpentine soils. Annu Rev Ecol Evol S 36: 243-266.

Cordell D, Rosemarin A, Schröder JJ, Smit AL (2011) Towards global phosphorus security: A systems framework for phosphorus recovery and reuse options. Chemosphere 84: 747-758.

Cordell D, Neset T-SS (2014) Phosphorus vulnerability: a qualitative framework for assessing the vulnerability of national and regional food systems to the multidimensional stressors of phosphorus scarcity. Global Environ Chang 24: 108–122.

Chappelka AH, Grulke NE (2016) Disruption of the ‘disease triangle’ by chemical and physical environmental change. Plant Biol 18: 5-12.

D’Andrea L, Rinaldi M (2010) Systems to evaluate the effects of atmospheric CO_2 concentration on field crops: a review of open top chambers. Italian Journal of Agrometeorology 1/2010: 23-34

Dong-Gyu K, Shi C, Watanabe M, Kita K, Satoh F, Koike T (2015) Growth of Japanese and hybrid larch seedlings grown under free-air O_3 fumigation—an initial assessment of the effects of adequate and excessive nitrogen. J Agr Meteorol 71: 239-244.

Eguchi N, Funada R, Ueda T, Takagi K, Hiura T, Sasa K, Koike T (2005) Soil moisture condition and growth of deciduous tree seedlings native to northern Japan raised under elevated CO_2 with a FACE system. Phyton 45: 133–138.

Eguchi N, Karatsu K, Ueda T, Funada R, Takagi K, Hiura T, Sasa K, Koike T (2008) Photosynthetic responses of birch and alder saplings grown in a free air CO_2 enrichment system in northern Japan. Trees 22: 437-447.

Harmens H, Mills G, Hayes F, Norris DA, Sharps K (2015) Twenty eight years of ICP vegetation: An overview of its activities. Ann Bot 5: 31-43.

Hill AC (1967) A special purpose plant environmental chamber for air pollution studies. J Air Pollut Cont Assoc 17: 743-748.

Hoshika Y, Carrari E, Zhang L, Carriero G, Pignatelli S, Fasano G, Materassi A, Paoletti E (2017) Testing a ratio of photosynthesis to O_3 uptake as an index for assessing O_3-induced foliar visible injury in poplar trees. Environ Sci Pollut Res Int. 25: 8113-8124.

Japan Meteorological Agency (2016) http://www.jma.go.jp/jma/indexe.html Website accessed on 8th January 2016

Karnosky DF, Zak DR, Pregitzer KS, et al. (2003) Tropospheric O_3 moderates responses of temperate hardwood forests to elevated CO_2: a synthesis of molecular to ecosystem results from the Aspen FACE project. Funct Ecol 17: 289-304.

Karnosky DF, Werner H, Holopainen T, et al. (2007) Free-air exposure systems to scale up ozone research to mature trees. Plant Biol 9: 181-190.

Kayama M, Choi D, Tobita H, Utsugi H, Kitao M, Maruyama Y, Nomura M, Koike T (2006) Comparison of growth characteristics and tolerance to serpentine soil of three ectomycorrhizal spruce seedlings in northern Japan. Trees 20: 430-440.

Kayama M, Satoh F, Koike T (2011) Photosynthetic rate, needle longevity, and nutrient contents in *Picea glehnii* growing on strongly acidic volcanic ash soil in northern Japan. Photosynthetica 49: 239-245.

Kayama M, Koike T (2015) Differences in growth characteristics and dynamics of elements in seedlings of two birch species grown in serpentine soil in northern Japan. Trees 29: 171-184.

Kim YS, Watanabe M, Imori M, Sasa K, Takagi K, Hatano R, Koike T (2011) Reduced atmospheric CH_4 consumption by two forest soils under elevated CO_2 concentration in a FACE system in northern Japan. J Japan Soc Atmos Environ 46: 30-36.

Kitao M, Komatsu M, Yazaki K, Kitaoka S, Tobita H (2015) Growth overcompensation against O_3 exposure in two Japanese oak species, *Quercus mongolica* var. *crispula* and *Quercus serrata*, grown under elevated CO_2. Environ Pollut 206: 133-141.

Kobayashi K (2015) FACE-ing the challenges of increasing surface ozone concentration in Asia. J Agr Met 71: 161-166.

Koike T, Watanabe M, Hoshika Y, Kitao M, Matsumura H, Funada R., Izuta T. 2013. In: Matyssek R, Clarke N, Cudlin P, Mikkelsen TN, Tuovinen J-P, Wieser G, Paoletti E (eds) Effects of ozone on forest ecosystems in East and Southeast Asia. Climate Change, Air Pollution and Global Challenges: Understanding and Perspectives from Forest Research. Elsevier, Oxford, 371-390.

Koike T, Watanabe M, Watanabe Y, Agathokleous E, Eguchi N, Takagi K, Satoh F, Kitaoka S, Funada R (2015) Ecophysiology of deciduous trees native to Northeast Asia grown under FACE (Free Air CO_2 Enrichment). J Agr Meteor 71: 174-184.

Macháčová K (2010) Open top chamber and free air CO_2 enrichment - approaches to investigate tree responses to elevated CO_2. iForest 3: 102-105.

Matsuda K, Shibuya M, Koike T (2002) Maintenance and rehabilitation of the mixed conifer-broadleaf forests in Hokkaido, northern Japan. Eurasian J For Res 5: 119-130.

Matyssek R, Clarke N, Cudlin P, Mikkelsen TN, Tuovinen J-P, Wieser G, Paoletti E. 2013. *Climate Change, Air Pollution and Global Challenges: Understanding and Perspectives from Forest Research*. Elsevier, Oxford. pp. 648.

Mcleod A, Shaw P, Holland M, (1992) The Liphook fumigation project: studies of sulphur dioxide and ozone effects on coniferous trees. Forest Ecol Manag 51: 121-127.

Menser HA, Heggestad HE (1964) Facility for ozone fumigation of plant materials. Crop Sci 4: 103.

Mills G, Buse A, Gimeno B, Bermejo V, Holland M, Emberson L, Pleijel H (2007) A synthesis of AOT40-based response functions and critical levels of ozone for agricultural and horticultural crops. Atmos Environ 41: 2630-2643.

Norby RJ, Zak DR (2011) Ecological Lessons from Free-Air CO_2 Enrichment (FACE) Experiments. Annu Rev Ecol Evol Syst 42: 181-203.

Nunn AJ, Reiter IM, Häberle K-H, Werner H, Langebartels C, Sandermann H, Heerdt C, Fabinan P, Matyssek R (2002) “Free-air” ozone canopy fumigation in an old-growth mixed forest: concept and observations in beech. Phyton 42: 105-119.

Okada M, Lieffering M, Nakamura H, Yoshimoto M, Kim HY, Kobayashi K (2001) Free-air CO_2 enrichment (FACE) using pure CO_2 injection: system description. New Phytol 150: 251-260.

Oksanen E (2001) Increasing tropospheric ozone level reduced birch (*Betula pendula*) dry mass within a five year period. Wat Air Soil Pollut 130: 947-952.

Paoletti E, Materassi A, Fasano G, Hoshika Y, Carriero G, Silaghi D, Badea O (2017) A new-generation 3D ozone FACE (Free Air Controlled Exposure). Sci Total Environ 575: 1407-1414.

Rafarel CR, Ashenden TW (1991) A facility for the large-scale exposure of plants to gaseous atmospheric pollutants. New Phytol 117: 345-349.

Reijnders L (2014) Phosphorus resources, their depletion and conservation, a review. Res Conserv Recy 93: 32-49.

Schmincke H-U (2004) Volcanism-Subduction zone volcanoes. New York: Springer-Verlag Berlin.

Shi C, Watanabe T, Koike T (2017) Leaf stoichiometry of deciduous tree species in different soils exposed to free-air O_3 enrichment over two growing seasons. Environ Exp Bot 138: 148-163.

Sicard P, Augustaitis A, Belyazid S (2016) Global topics and novel approaches in the study of air pollution, climate change and forest ecosystems. Environ Pollut 213: 977-87.

Takeda M, Komatsu H. 2007. Development of a comprehensive monitoring method for assessing the vegetation decline at beech forest region. National Institute for Environmental Studies, Japan, Code: 0509AH953, 71-76. (In Japanese)

Tang H, Liu G, Han Y, Zhu J, Kobayashi K (2011) A system for free-air ozone concentration elevation with rice and wheat: Control performance and ozone exposure regime. Atmos Environ 45: 6276-6282.

Uemura S. 1992. Environmental factors controlling the distribution of forest plants with special reference to floral mixture in the boreo-nemoral ecotone, Hokkaido island. Environ. Sci. Hokkaido University 15: 1-54.

Unsworth MH, Heagle AS, Heck WW (1984) Gas exchange in open-top field chambers-I. Measurement and analysis of atmospheric resistances to gas exchange. Atmos Environ 18: 373-380.

Velikova V, Tsonev T, Pinelli P, Alessio GA, Loreto F (2005) Localized ozone fumigation system for studying ozone effects on photosynthesis, respiration, electron transport rate and isoprene emission in field-grown Mediterranean oak species. Tree Physiol 25: 1523-1532.

Wang XN, Agathokleous E, Qu L, Watanabe M, Koike T (2016) Effects of CO_2 and/or O_3 on the interaction between root of woody plants and ectomycorrhizae. J Agr Meteorol 72: 95-105.

Watanabe M, Ryu K, Kita K, Takagi K, Koike T (2012) Effect of nitrogen load on growth and photosynthesis of seedlings of the hybrid larch F_1 (*Larix gmelinii* var. *japonica* × *L. kaempferi*) grown on serpentine soil. Environ Exp Bot 83: 73-81.

Watanabe M, Mao Q, Novriyanti E, Kita K, Takagi K, Satoh F, Koike T (2013a) Elevated CO_2 enhances the growth of hybrid larch F_1 (*Larix gmelinii* var. *japonica* × *L. kaempferi*) seedlings and changes its biomass allocation. Trees 27: 1647-1655.

Watanabe M, Hoshika Y, Inada N, Wang X, Mao Q, Koike T (2013b) Photosynthetic traits of Siebold's beech and oak saplings grown under free air ozone exposure in northern Japan. Environ Pollut 174: 50-56.

Werner H, Fabian P (2002) Free-air fumigation of mature trees: A novel system for controlled ozone enrichment in grown-up beech and spruce canopies. Environ Sci Pollut Res 9: 117-121.

Wulff A, Hänninen O, Tuomainen A, Kärenlampi L (1992) A method for open-air exposure of plants to ozone. Ann Bot Fennici 29: 253-262.

Zhang L, Hoshika Y, Carrari E, Burkey KO, Paoletti E (2017) Protecting the photosynthetic performance of snap bean under free air ozone exposure. J Environ Sci. In Press. 66: 31-40.

Zheng SJ (2010) Crop production on acidic soils: overcoming aluminum toxicity and phosphorus deficiency. Ann Bot 106: 183-184.

Table 1

Monthly mean air temperature, wind speed and relative humidity and monthly total sunshine duration and precipitation at Sapporo, Japan, for the months May-November of the experimental years 2014-2015. For each year, the median ±MAD of the monthly values are presented. MAD denotes the median absolute deviation, which is the median of the absolute deviations from the data median: $MAD = median_i\left(\left|X_i - median_j\left(X_j\right)\right|\right)$.

	May		June		July		August		September		October		2014	2015
	**2014**	**2015**	**2014**	**2015**	**2014**	**2015**	**2014**	**2015**	**2014**	**2015**	**2014**	**2015**		
**Daily air temperature (^{o}C)**	14.0	14.2	18.7	16.7	22.5	21.3	22.4	22.4	18.1	18.4	11.3	10.8	18.4±4.1	17.6±3.6
**Daily wind speed (m s^{-1})**	4.1	4.4	4.0	3.7	3.4	3.1	3.1	3.0	3.3	2.8	3.2	4.0	3.35±0.2	3.4±0.5
**Daily relative humidity (%)**	65	56	73	73	70	73	73	73	68	71	64	61	69±4	72±1
**Total sunshine duration (h)**	207.7	263.2	182.4	151.4	214.3	175.0	178.9	158.6	188.8	151.8	145.4	150.9	185.6±14.4	155.2±4.1
**Total precipitation (mm)**	60.0	37.0	99.0	66.5	76.5	64.0	217.5	131.5	146.0	198.0	124.0	98.0	111.5±34.8	82.3±31.8
